# Jevons Paradox: Rise of the Machines May Create More Work for Physicians

**DOI:** 10.1161/SVIN.126.002321

**Published:** 2026-03-03

**Authors:** Jens Fiehler

**Affiliations:** Department of Diagnostic and Interventional Neuroradiology, University Medical Center Hamburg-Eppendorf, Germany.

**Keywords:** Editorials, artificial intelligence, economics, radiology, robotics, telemedicine

It is a confusion of ideas to suppose that the economical use of fuel is equivalent to diminished consumption. The very contrary is the truth.^1^

Stanley Jevons (1865)

The surprising thing is the prediction that radiologists would be the first jobs to go was exactly the opposite. The trend shows that more radiologists are being hired now as a result of AI.

Jensen Huang, *CEO*, *Nvidia*, November 19, 2025, U.S.-Saudi Investment Forum^2^

Both citations, separated by 160 years, describe a strikingly similar phenomenon that seems to apply to both steam engines and the latest technological developments, such as artificial intelligence (AI) and robotics: the Jevons paradox.

Almost a decade ago, it was predicted in an *New England Journal* editorial that “...machine learning will displace much of the work of radiologists and anatomic pathologists. These physicians focus largely on interpreting digitized images, which can easily be fed directly to algorithms instead...”^3^ The idea of humans being replaced by machines dates back to the Industrial Revolution, which gave rise to Luddite slogans (Death to the machines) and the word sabotage. The term is connected to workers throwing wooden shoes (French: sabot) into machinery to destroy it out of fear of losing their jobs. However, replacement by machines has not led to long-term unemployment on a societal level.

Is it worth sabotaging AI and robotics to resist our replacement? Physicians might support unnecessary bureaucratic obstacles to the deployment of AI algorithms and robotics and use ethical concerns as a pretext to postpone their adoption, rather than out of genuine worry about ethics. In fact, we see no signs of displacement of radiologists by AI. There are numerous explanations. “Tasks are not jobs” is a preeminent one. Another one is that there is simply an ever-increasing number of imaging exams ordered, with not much actual AI involvement as of now.

The Jevons paradox explains more broadly why efficiency gains by technology may not necessarily reduce workload or costs but instead might increase them. It is named after the English economist and logician William Stanley Jevons (1835–1882). He introduced the paradox in his book, The Coal Question, where he observed that technological improvements in the efficiency of steam engines increased overall coal consumption in Britain rather than reducing it, as lower costs spurred greater demand and usage.

This phenomenon occurs because efficiency gains decrease the marginal cost of using a resource, which, in turn, stimulates greater demand as consumers expand usage of the resource. The usage expands until the shrinking marginal benefit of using the resource (one more time) exceeds the reduced marginal costs of using the resource (one more time). A new equilibrium emerges where efficiency gains lead to higher total costs because of the higher number of units used, even if the individual resource use becomes cheaper. As a third-order effect, the resulting overall market growth attracts further investment in technological development and accelerates this phenomenon again. Basically, it is about the imbalance of cost and benefit until a new balance emerges. Understanding the underlying forces is difficult because the price tag is only partially measurable in dollars.

Early signals suggest a Jevons-like rebound effect for radiology. As AI will lower the marginal cost of interpreting another imaging study (in time, cognitive effort, and perceived risk), the perceived threshold for ordering another imaging exam will drop. Emergency physicians feel more comfortable ordering computed tomography scans if turnaround times are shorter and diagnostic confidence is higher. The result? Same or even more radiologists at work.

Radiology has experienced a similar pattern before. The transition from film to digital Picture Archiving and Communication System dramatically improved efficiency and accessibility, but it also coincided with explosive growth in imaging volume. A tool, intended to help radiologists do more with less may instead lead to doing vastly more with a bit more, with the total interpretive burden continuing to rise.

There is also a subtler phenomenon at play. Once AI automates routine tasks, radiologists’ time may be freed for more complex cases, integrate nonimaging information, multidisciplinary collaboration, and advanced imaging techniques. While professionally enriching, these activities often generate additional imaging demand. Thus, AI may elevate the cognitive load for radiology work while simultaneously expanding its volume.

With clinical application still in its infancy, a parallel story is unfolding in neurointerventions. Robotic systems are emerging to assist with thrombectomy, aneurysm coiling, and flow diverter placement.^4,5^ Compared with human operators, robots promise greater precision, reduced operator fatigue, lower radiation exposure, and even allow completely new, more complex procedures. The most provocative robotic promise is the potential for remote intervention and, thus, scaling from individual craftsmanship to industry production. Like with AI, efficiency and capability gains will drive increased utilization.

When neurointerventional robots were introduced to early adopters, the purchasing cost was granted by administrators who wanted to cut a red ribbon in front of the camera of the local TV station. When reality kicked in, it became apparent that robots did not necessarily increase the efficiency of neurointerventions, even if navigation and device deployment looked good. The overall procedure duration is determined by much more: by opening packages and inserting catheters, wires, and devices. This often took longer than just handing them to an experienced interventionalist.

Robots will have their breakthrough as soon as they perform complex interventions and enable less-experienced operators safely without delaying total procedure times. Then, the indication threshold may shift. Patients previously deemed marginal candidates may then be treated. Hospitals without elite specialists may adopt robotic platforms to expand their procedural offerings. Regional or even national telestroke and teleneurointervention networks may emerge, dramatically improving access and elevating numbers. In this scenario, technological efficiency increases the volume of interventions. More patients are treated, more procedures are performed, more devices are deployed, with more downstream imaging, follow-up, and retreatment.

The cap in neurovascular robotics appears to depend on disease incidence, which could be considered a major rate-limiting step affecting the demand for more physicians. However, by lowering procedural risks, it may become beneficial to operate on earlier or less severe disease states, along with entirely new indications, thereby increasing the overall number of surgeries.

Looking through the lens of the business classic, The Innovator’s Dilemma,^6^ robotic neurointerventions initially appear as technology for top-tier academic centers: expensive, complex, and optimized for high-end performance. Incumbent neurointerventional programs, focused on the most complex cases treated by highly skilled operators, may underinvest in early robotic adoption, only to find that the technology later redefines the competitive landscape by decentralizing expertise. Over time, however, robotic platforms are likely to move down-market. As systems become cheaper, more automated, and easier to use, they could enable capable but less specialized centers to perform procedures once confined to elite institutions. The strategic risk for incumbents is 2-fold: losing procedural share to distributed, robot-enabled networks, and underestimating how efficiency gains can fuel demand growth (Jevons again).

Each success reinforces adoption; each new indication expands demand. Just as laparoscopic surgery led to more surgeries overall by reducing recovery time and perceived risk,^7^ robotic neurointerventions will likely increase the total procedural volume with more patients and additional indications rather than replace existing approaches one-for-one.

If we focus on patient benefit, AI and robotics will help improve health care. Before contemplating slowing down their progress for cost reasons, we should examine how other attempts at optimization have led to cost expansion based on another principle: Parkinson Law on Administration.^8^ Instead of following price and demand dynamics like the Jevons paradox, it explains that administration tends to grow steadily by 5% to 7% annually, independent of the actual demand and workload, whether stable, increasing, or decreasing.

Parkinson law states that work expands to fill the time available for its completion. Demand for growth is not coming from customer needs but from administrators who generate internal tasks to justify their existence, create subordinate roles, and multiply paperwork. Half-ironically, Parkinson presented the growth as a mathematical equation, with the formula *x=*(*2k*^*m*^*+P*)*/n,* in which *k* was the number of officials wanting subordinates and *m* was the hours they spent writing minutes to each other. I think some of us have encountered this phenomenon in one form or the other.

Machines will not replace us (unless you expect artificial general intelligence will take over). According to Jevons, efficiency gains from new technologies such as AI and robotics are more likely to lead to an increase in workload rather than a reduction. They will raise overall health care costs instead of lowering them. Ultimately, the question is not whether AI and robotics will increase our workload, they almost certainly will, but whether that increase will align with patient benefit.

## ARTICLE INFORMATION

### Disclosures

Dr Fiehler is a consultant for Acandis, Cerenovus, Medtronic USA, Inc, MicroVention, Inc, Penumbra, Inc, Phenox, Philips, Roche, Stryker Corp, Tegus Medical, and Tonbridge, and is the Managing Director for Eppdata.
